# Transcriptome analysis and gene expression analysis related to salinity-alkalinity and low temperature adaptation of *Triplophysa yarkandensis*

**DOI:** 10.3389/fgene.2022.1089274

**Published:** 2023-01-12

**Authors:** Xuejing Zhang, Tai Wang, Dongdong Zhai, Hongyan Liu, Fei Xiong, Ying Wang

**Affiliations:** ^1^ Hubei Engineering Research Center for Protection and Utilization of Special Biological Resources in the Hanjiang River Basin, Jianghan University, Wuhan, China; ^2^ Gansu Key Laboratory of Cold Water Fishes Germplasm Resources and Genetics Breeding, Gansu Fishers Research Institute, Lanzhou, China; ^3^ Hubei Key Laboratory of Environmental and Health Effects of Persistent Toxic Substances, Jianghan University, Wuhan, China

**Keywords:** *T. yarkandensis*, transcriptome, gene annotation, salinity-alkalinity adaptation, low temperature adaptation

## Abstract

*T. yarkandensis* is a common species of *Triplophysa*, and it is distributed in Shule river of Hexi Corridor, of Gansu province in China. In order to enrich gene database resources and explore the environment adaptation of *T. yarkandensis*, fifteen tissues were collected from three adult *T. yarkandensis* for transcriptome sequencing and *de novo* assembly. Nine major international gene annotation databases (NR, COG, egg_NOG, TrEMBL, Pfam, KOG, Swiss prot, KEGG and Gene Ontology) were utilized to annotate unigenes. A detailed study was conducted to explore the gene expression and the differentially expressed genes among five tissues (brain, heart, kidney, liver and spleen). In addition, the current study showed that candidate genes involved in salinity-alkalinity and low temperature adaptation were differentially expressed in tissues of *T. yarkandensis*. Precisely, *mapk1*, *abcc1*, *gpx1*, *gpx4*, *cat* and *aqp1* genes participated in the regulation process of salinity-alkalinity adaptation, and *elovl4*, *acaca*, *fasn*, *acaa2*, *acox1* and *acox3* genes were involved in fatty acid metabolism and closely associated with low temperature adaptation. On the one hand, it was found that the expression of these genes varied among different tissues, and the important pathways involved in these genes were mapped. Furthermore, we analyzed *mapk1* and *acox1* genes in depth to obtain the predicted gene structure and important amino acid sites. The transcriptome information in this study will be conducive to provide further understanding for the molecular level research and exploration of the environmental adaptation of *T. yarkandensis*.

## Introduction

*T. yarkandensis* belongs to the *Triplophysa* genus within the Nemacheilidae family in the order Cypriniformes (Chen et al., 2017), and it is the most respresentative fish species of the fish population in the adjacent Qinghai-Tibetan Plateau and one of the high-altitude fishes (Chen et al., 2010). *T. yarkandensis* has a length and weight of about 30.0 cm and 305 g respectively (Chen et al., 2017), it possess the biological characteristics of benthic life, with periodic migration, selective spawning, and fierce feeding (Chen and Yao, 2008). The optimal physiological function for *T. yarkandensis* are encountered at a temperature of 6°–7°C, which means that this fish species has a strong low temperature adaptability (Xiang and Fan, 2014). Shule river is one of the three major inland rivers in Gansu province of China. It is located at the westernmost end of Hexi Corridor, with temperature of 6.98°C–9.82°C (Wang et al., 2022a). Shule river mainly came from melting snow and ice, atmospheric precipitation and groundwater (Guo et al., 2015). In line with the research conducted on the hydrochemical characteristics of Shule river basin, the main ions were Ca^2+^, Mg^2+^, Na^+^, HCO_3_^−^, SO_4_^2-^, Cl^−^. The river shows a bicarbonate alkalinity with a pH of 7–8, which indicates a weak alkaline (Zhou et al., 2004; Yang et al., 2021).

In recent years, in view of the sharp decline of natural resources of *T. yarkandensis*, researchers have started to focus on the characteristics of *T. yarkandensis* population (Chen et al., 2011) and reproduction (Chen et al., 2013). Additionally, the complete mitochondrial genome of *T. yarkandensis* has been determined (Chen et al., 2016). Besides, a research to develop microsatellite markers of the populations of *T. yarkandensis* showed that the microsatellite markers of dibasic repeat was significantly in *T. yarkandensis* (Wang et al., 2020). Furthermore, the assessment of the otolith morphology was applied for the first time to classify and identify the population of *T. yarkandensis*. This analysis found that the relationship between otolith morphology and fish body growth greatly reflected the adaptability of the individuals’ development to their habitat (Wang et al., 2022b). Also, a study on genetic diversity of different geographical populations based on mitochondrial *Cytb* and D-loop sequences indicated that *T. yarkandensis* populations in Tarim river basin were relatively stable and no obvious population expansion occurred recently (Zhao et al., 2022).

In recent years, RNA sequencing (RNA-Seq) has been prevalently used in fundamental research as an effective tool and standard for transcriptome analysis (Dewey and Li, 2011). Transcriptome studies could comprehensively elucidate the functions and structures of genes and the molecular mechanisms of biological processes (Guan et al., 2020). As revealed by the transcriptome analysis of *Leuciscus waleckii*, genes involved in ion transportation was crucial in the acid-base homeostasis of fish under alkaline stress (Chang et al., 2014). Transcriptomic data identified several immune-related genes in *Betta splendens* (Amparyup et al., 2020). Transcriptome analysis of *Triplophysa dalaica* identified genes that were involved in hypoxia response (Wang et al., 2015). Liver transcriptome analysis not only identified that genes were involved in the responses of turbot to different salinity conditions, but also demonstrated that low salinity exposure disorders turbot lipid metabolism (Liu et al., 2020). Transcriptomic and metabolomic analyses indicated that when compared with diploid *Apostichopus japonicus*, triploid *A. japonicus* had more advantages in immunity, growth and development, stress resistance and adaptability to a harsh living environment (Xie et al., 2022). Based on RNA-seq, comparative transcriptome analysis of different tissues in *Lota dalliance* provided a reference for enriching gene resource (Yang et al., 2020). At present, studies have used the gill tissue of *T. yarkandensis* to analyze the comparative transcriptome of *T. yarkandensis* in response to salinity and alkalinity stress (Chen et al., 2020). However, there are no related studies that have been reported on multi-tissue transcriptome analysis and environmental adaptation of *T. yarkandensis.*

In the current study, the samples of brain, heart, kidney, liver and spleen of *T. yarkandensis* were extracted to provide a more comprehensive transcriptome reference. In addition, candidate genes involved in salinity-alkalinity and low temperature adaptation from the transcriptome of *T. yarkandensis* were selected. The results from the current study will provide additional knowledge on the biology of *T. yarkandensis* which can be used to improve conservation and breeding practices of this fish species.

## Materials and methods

### Sample collection, RNA extraction and sequencing

Three adults of *T. yarkandensis* were collected from the reach of Shule river basin (96.71 E, 40.57 N) at an altitude of 1,300 m. A total of fifteen tissues were collected from the three fish specimens including heart, liver, brain, spleen and kidney. These tissues were stored in ultra-low temperature liquid nitrogen at −80°C for standby. Liquid nitrogen was added to grind the tissues into powder, which allowed to extracted total RNA from the mixed tissues using the standard Trizol kit (Promega SV Total RNA Isolation system), and detect the quality of total RNA using agarose gel electrophoresis. This process allowed, the detection of the degradation degree, purity, quality, concentration and integrity of total RNA by Nanodrop spectrophotometer (Thermo Fisher Scientific, MA, United State), Qubit fluorescence quantitative analyzer (Thermo Fisher Scientific, MA, Unite State) and Agilent 2,100 biological analyzer (Agilent, CA, United State), to ensure that the samples met the requirements for transcriptome sequencing. After detection, the mRNA within the total RNA was broken into specific short fragments, and the double-terminal cDNA library of the transcriptome was amplified by RT-PCR. Based on sequencing by synthesis (SBS) technology, the cDNA library was sequenced by Illumina Hiseq high-throughput sequencing platform (Illumina, CA, United State), which could produce a large number of high-quality reads (raw data). After detecting quality and filtering, clean reads were produced.

### De novo assembly and functional annotation

The clean reads were employed for *de novo* assembly using Trinity (v2.5.1) (Grabherr et al., 2011). The CD-HIT-EST program was utilized to remove the redundant sequences, and the parameters were set as the similarity of 0.95 and the sequence length of 10 bases (Li and Godzik, 2006). Annotation information were finally obtained by selecting BLAST (Altschul et al., 1997) parameter E-value ≤ 1e-5 and Hmmer (Finn et al., 2014) parameter E-value ≤ 1e-10. The annotation information of unigenes were derived from nine databases, including NCBI non-redundant protein sequence (NR) (Deng et al., 2006), Swiss prot (Rolf et al., 2004), Clusters of Orthologous Groups (COG) (Tatusov et al., 2000), EuKaryotic Orthologous Groups (KOG) (Koonin et al., 2004), Evolutionary genealogy of genes: Non-supervised Orthologous Groups (eggNOG) (Jaime et al., 2016), Gene Ontology (GO) (Ashburner et al., 2000), Kyoto Encyclopedia of Genes and Genomes (KEGG) (Minoru et al., 2004), Protein family (Pfam) (Finn et al., 2014), and Translation of EMBL (TrEMBL) (Amos and Rolf, 2000).

### Gene expression and differential expression genes (DEGs) analysis

Bowtie (Langmead, 2009) was applied to compare the sequenced reads with the unigene library, and the expression level was estimated in combination with RSEM (Dewey and Li, 2011). The expression abundance of corresponding unigene was expressed by FPKM (Fragments Per Kilobase of transcript per Million mapped reads) value (Trapnell et al., 2010). Differential expression analysis was performed using DeSeq2 (v1.6.3) (Love et al., 2014). Differential expression results were obtained with FDR (False Discovery Rate) > .01 and the difference multiple FC (fold change) ≥ 2 were considered as the screening standards. The differentially expressed genes of five tissues were analyzed by GO functional annotation and KEGG pathway enrichment analysis.

### Scanning for candidate genes in the salinity-alkalinity adaptation

In Shule river, the cations were predominantly Ca^2+^, Mg^2+^, Na^+^ and the anions were mainly HCO_3_^−^, SO_4_^2-^, Cl^−^, and the pH was 7–8 (Zhou et al., 2004). The water was weakly saline and alkaline. Studies have indicated that *T. yarkandensis* had a certain tolerance under precondition of salinity and alkalinity stress (Yao et al., 2018). In the current study, six genes were identified which participated in the regulation process of salinity-alkalinity adaptation, these genes were *mapk1* (mitogen-activated protein kinase 1), *abcc1* (ATP-binding cassette sub-family C member 1), *gpx1* (glutathione peroxidase 1), *gpx4* (glutathione peroxidase 4), *cat* (catalase) and *aqp1* (aquaporin-1) (Li et al., 2016; Chen et al., 2020; Zhang, 2020; Wang et al., 2022c). From the differential genes obtained in this study, the six genes were searched and the differences between tissues were analyzed. Additionally, according to the results of KEGG enrichment, the pathway map involving these genes was obtained. Finally, *mapk1* gene was selected for bioinformatics analysis, including sequence characteristics and important amino acid sites. Firstly, FGENESH (http://www.softberry.com/berry.phtml?topic=fgenesh&group = programsandsubgroup = gfind) was used to analyze its sequence characteristics. Moreover, a search at the BLAST tool at NCBI was conducted for similar sequences of *mapk1* gene, and important amino acid sites were compared by using Clustal Omega (https://www.ebi.ac.uk/Tools/msa/clustalo/).

### Scanning for candidate genes in fatty acid metabolism related to low temperature adaptation

Water temperature is one of the crucial environmental factors not only affecting almost all life activities of fish, but also affecting the physiological and biochemical reactions of fish. Fish can gradually adapt low temperature environment by regulating the concentration of enzymes in different metabolic pathways. A previous report not only indicated that low temperature heightened lipid synthesis in the body of *Trilophysa bleekeri*, but also showed the content of polyunsaturated fatty acids in the body was up-regulated in the low temperature group (Yuan, 2021). Through fatty acid metabolism, the content of long-chain unsaturated fatty acids in phospholipid of cell membrane increased and the cell membrane would maintain stability and fluidity. In the current study, six genes was identified, which are involved in fatty acid metabolism pathway, these genes were *acox1* (acyl-CoA oxidase 1, palmitoyl), *acox3* (acyl-CoA oxidase 3, pristanoyl), *acaa2* (acetyl-CoA acyltransferase 2), *elovl4* (elongation of very long chain fatty acids elongase gene 4), *acaca* (acetyl-CoA carboxylases alpha) and *fasn* (fatty acid synthase) (Xu et al., 2020; Yang et al., 2020; Wang et al., 2022d; Liu et al., 2022). From the differential genes obtained in this study, the six genes were searched and the differences among tissues were analyzed. Moreover, according to the results of KEGG enrichment, the pathway map involving these genes was obtained. Finally, *acox1* gene was selected for bioinformatics analysis, including sequence characteristics and important amino acid sites. Firstly, FGENESH (http://www.softberry.com/berry.phtml ?topic = fgeneshandgroup = programsandsubgroup = gfind) was used to analyze its sequence characteristics. Moreover, a search at the BLAST tool at NCBI was conducted for similar sequences of *acox1* gene, and important amino acid sites were compared by using Clustal Omega (https://www.ebi.ac.uk/Tools/msa/clustalo/).

## Results

### Sequencing, *de novo* assembly and functional annotation

The cDNA library was sequenced using Illumina Hiseq high-throughput sequencing platform. After sequencing quality control, a total of 102.11 Gb clean data were obtained. Overall, the percentage of Q30 bases of all products exceeded 91.28%, and the GC content exceeded 44.90%. The sample sequencing data evaluation statistics information was shown in the Supplementary Table S1. A total of 100,112 unigenes were obtained by assembly, the N50 of transcript and unigene were 3,938 bp and 2,579 bp, with high assembly integrity (Supplementary Table S2, Supplementary Figure S1).

**FIGURE 1:**
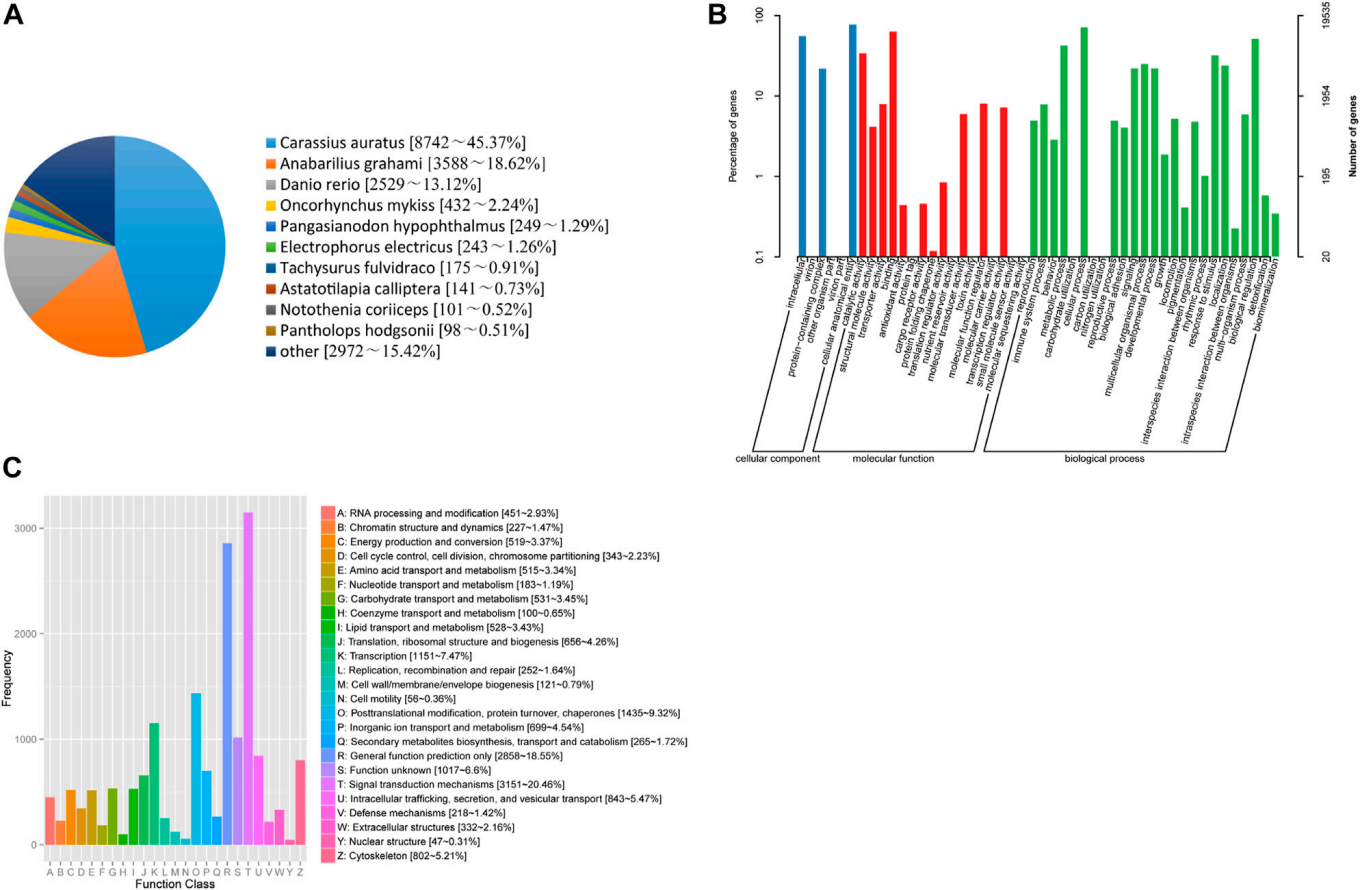
**(A)** Species distribution results of a similarity search of unigenes against Nr databases. **(B)** Functional classification of assembled unique sequences based on GO terms. **(C)** Functional classification of assembled unigenes based on KOG.

In the current study, 23,008 unigenes were annotated to nine databases, including 5,207, 19,535, 19,637, 15,404, 18,696, 13,941, 19,259, 18,421 and 19,270 unigenes in COG, GO, KEGG, KOG, Pfam, Swiss prot, TrEMBL, eggNOG and NR databases, respectively (Supplementary Table S3). Based on the BLASTx similarity analysis against the NR database, *T. yarkandensis* unigenes had the highest number of hits compared to other fish species such as to *Carassius auratus* (45.37%), followed by *Anabarilius grahami* (18.62%), *Danio rerio* (13.12%) (Figure 1A). A total of 19,637 assembled unigenes were assigned to 48 terms for GO classification in the biological process (25 terms), cellular component (6 terms), and molecular function (17 terms) categories (Figure 1B). Cellular anatomical entity (15,116 unigenes) showed the most annotated unigenes, followed by cellular process (13,879 unigenes), binding (12,320 unigenes), and intracellular (10,866 unigenes). In KOG database (Figure 1C), unigenes have been divided into 25 functional categories, and among them, the largest was signal transduction mechanisms, with 3,151 unigenes, accounting for 20.46%, followed by nothing but general function prediction (2,858 unigenes, 18.55%), posttranslational modification, protein turnover, chaperones (1,435 unigenes, 9.32%). No unknown genes were found from the KOG database annotation.

### Analysis of differentially expressed genes (DEGs) among five tissues

**FIGURE 2:**
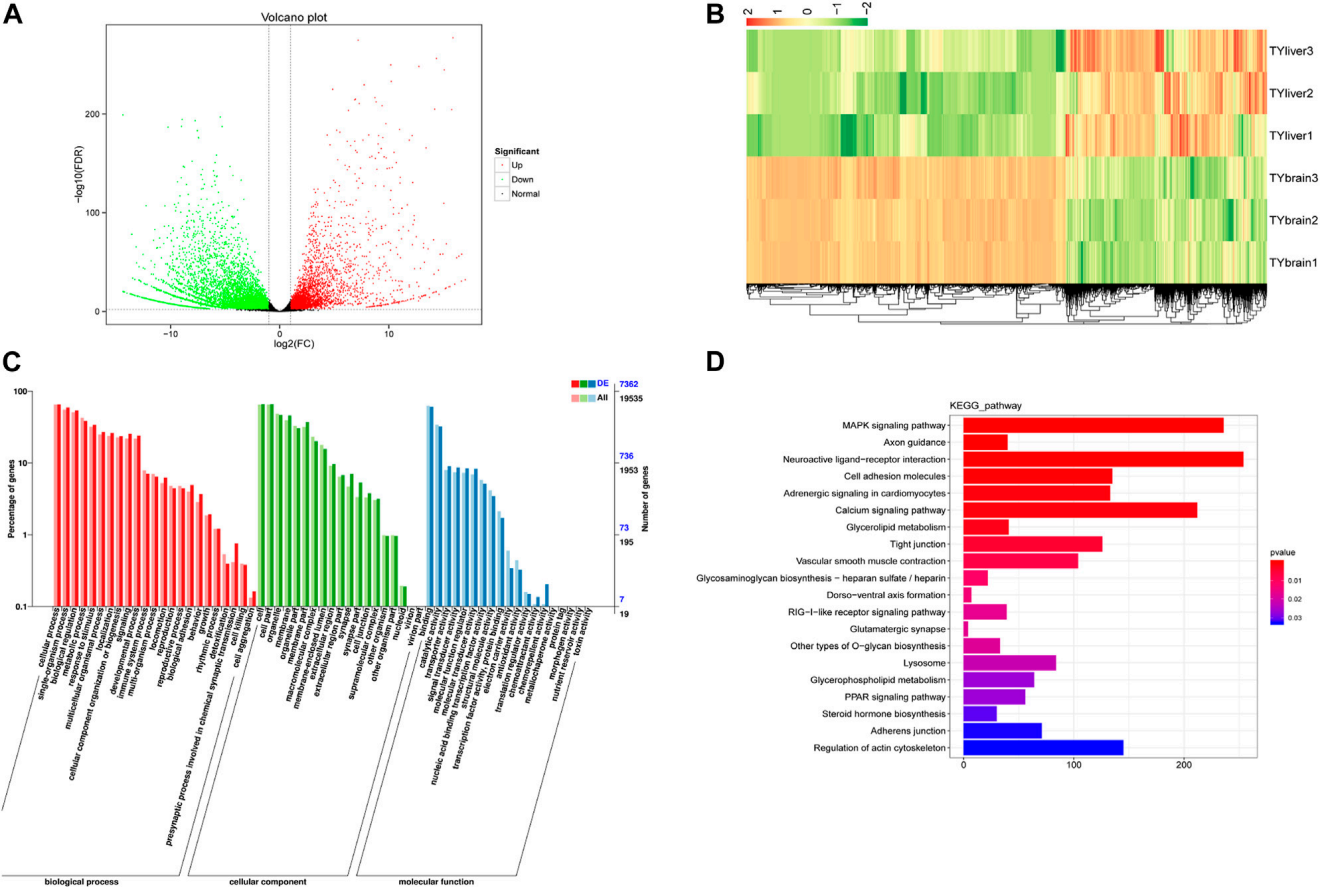
Differentially expressed genes (DEGs) analysis between brain and liver of *T. yarkandensis*. **(A)** Volcano plot of DEGs between brain and liver. Each point represents a gene. The green and red dots in the figure represent significantly differentially expressed genes, with green representing down-regulated gene expression, red representing up-regulated gene expression, and black dots represents a gene with no significant difference in expression. **(B)** Hierarchical clustering analysis was performed on the DEGs between brain and liver. The color represents log_2_(FC). **(C)** GO classification of DEGs between brain and liver. **(D)** KEGG enrichment of DEGs between brain and liver.

We compared DEGs in five tissues: brain, heart, liver, kidney and spleen. Results of differentially expressed genes between each two tissues were shown in Supplementary Table S4. TYbrain_vs_TYliver screened the largest differentially expressed genes number, with a 9,814 DEGs, of which 3,818 genes were up-regulated and 5,996 genes were down-regulated (Figure 2A). Hierarchical clustering analysis was performed on the selected differentially expressed genes between brain and liver, and the genes with the same or similar expression behavior were clustered (Figure 2B). GO annotation results and KEGG enrichment of DEGs between brain and liver were shown in Figures 2C, D. The three biological duplicate samples of the same tissue could cluster, indicating that the biological duplicate of the samples used in this study was favorable. The GO annotation results of ten groups were similar. Most of the terms included “cellular process”, “single-organism process”, “biological regulation”, “metabolic process”, “cell”, “cell part”, “binding” and “catalytic activity”. In KEGG pathway enrichment analysis, DEGs were enriched including MAPK signaling pathway, calcium signaling pathway and neuroaction ligand-receptor interaction.

### Signal pathway analysis was performed by KEGG

19,637 unigenes in KEGG database correspond to 330 pathways, and the top 20 pathways with the largest number of sequences were shown in the Supplementary Table S5. Unigenes with the largest three pathways annotations (neuroactive ligand-receptor interaction, calcium signaling pathway and MAPK signaling pathway) were associated with environmental information processing. In addition, eight of the top 20 pathways were correlated with signaling molecules and interaction, signal transduction. A previous study showed that different selenium sources could regulate the adaptation of yellow catfish to low temperature stress, neuroactive ligand-receptor interaction pathway significant enrichment (Hu et al., 2021). In the current study, the number of unigenes annotated in the neuroactive ligand-receptor interaction pathway was the largest, which was speculated to be associated with the low temperature environment adaptation of *T. yarkandensis*.

### Candidate genes involved with salinity-alkalinity adaptation

**FIGURE 3:**
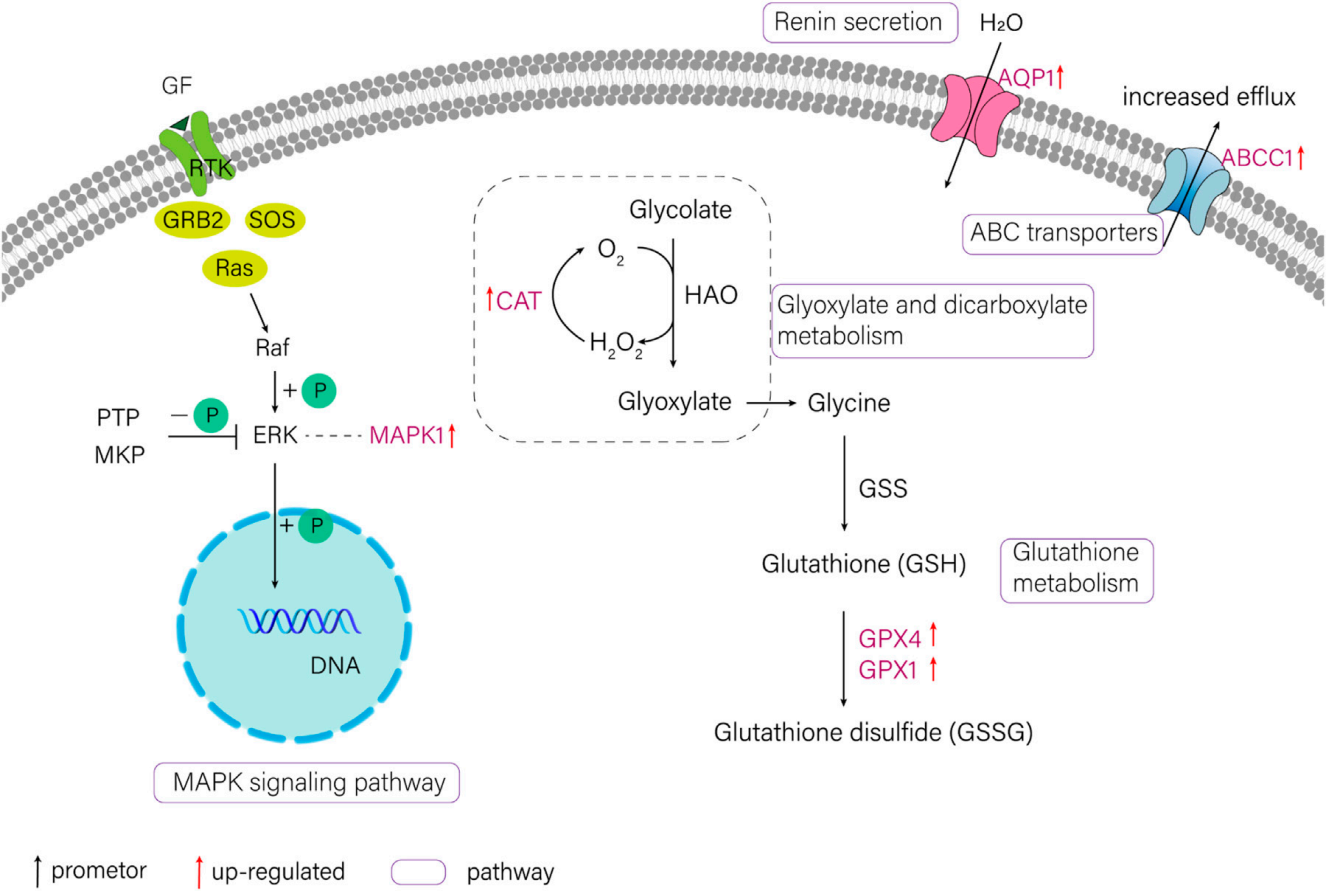
The schematic diagram of several pathways about salinity-alkalinity adaptation of *T. yarkandensis*. CAT (catalase), GPX1 (glutathione peroxidase 1), GPX4 (glutathione peroxidase 4), MAPK1 (mitogen-activated protein kinase 1), AQP1 (aquaporin-1), ABCC1 (ATP-binding cassette, subfamily C (CFTR/MRP), member 1).

**FIGURE 4:**
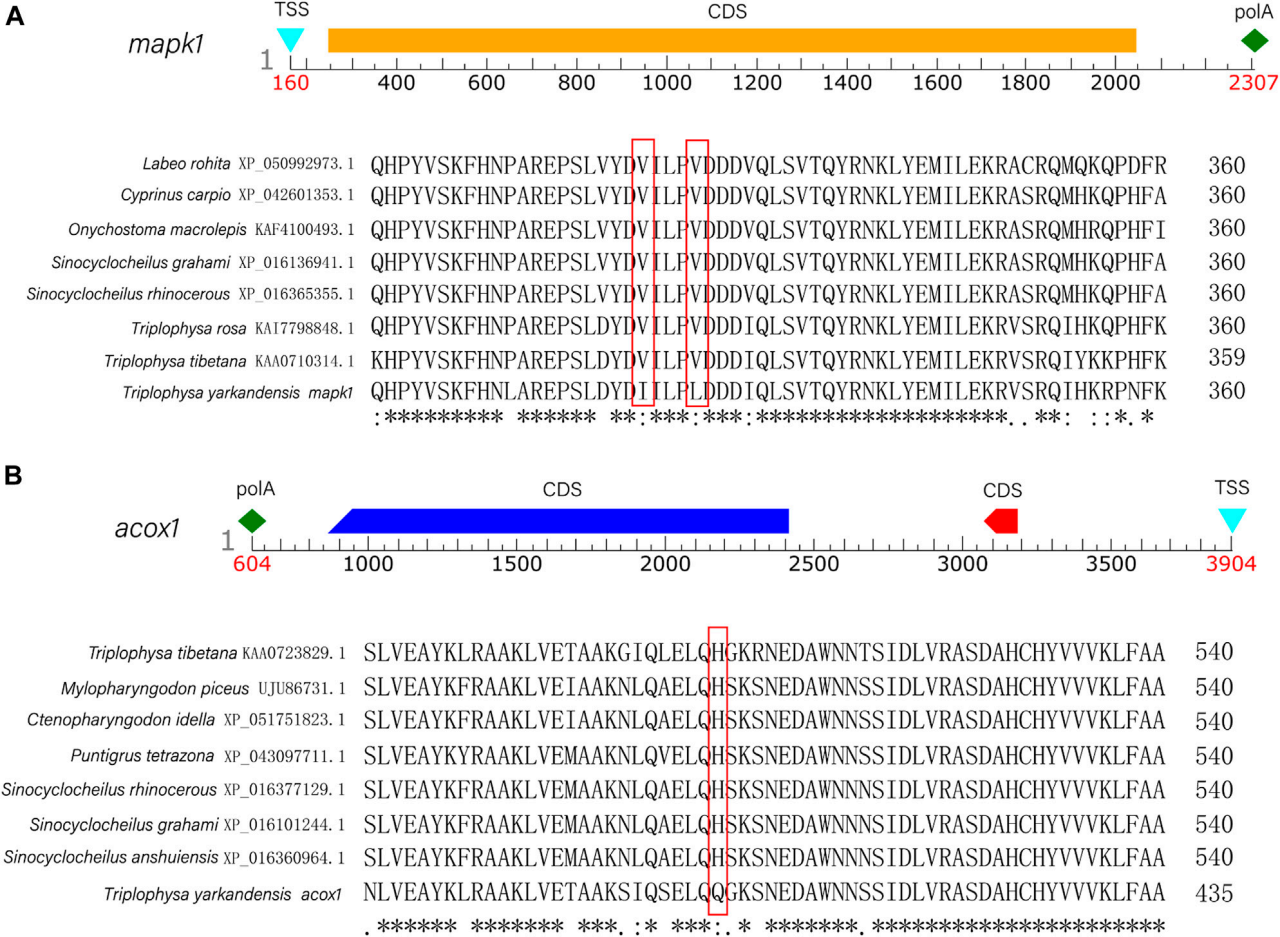
**(A)** The predicted sequence structure and a comparison of homologous the amino acid sequences with seven other fish (*Triplophysa rosa*, *Triplophysa tibetana*, *Sinocyclocheilus grahami*, *Sinocyclocheilus rhinocerous*, *Cyprinus carpio*, *Labeo rohita*, *Onychostoma macrolepis*) of *mapk1* gene. **(B)** The predicted sequence structure and a comparison of homologous the amino acid sequences with seven other fish (*Triplophysa tibetana*, *Mylopharyngodon piceus*, *Ctenopharyngodon idella*, *Puntigrus tetrazona*, *Sinocyclocheilus rhinocerous*, *Sinocyclocheilus grahami*, *Sinocyclocheilus anshuiensis*) of *acox1* gene.

In the current study, six candidate genes in the salinity-alkalinity adaptation: *mapk1*, *abcc1*, *cat*, *gpx1*, *gpx4* and *aqp1* were screened, and the expression differences of six genes among five tissues of *T. yarkandensis* were analyzed*.* The experimental results indicated that these genes were differentially expressed among the five tissues. As shown in Table 1, the expression of *cat*, *abcc1* and *gpx4* genes in liver was higher than that in brain, heart, kidney and spleen, while the expression of *gpx1* and *aqp1* genes in liver, heart, kidney and spleen was higher than that in brain. In addition, the expression of *mapk1* genes in liver and kidney was higher. A previous research revealed that salinity stress could affect the changes of metabolic pathways (Liu et al., 2020). The six genes involved in the pathway were shown in Figure 3. Six genes in five tissues of *T. yarkandensis* were differentially expressed and all were highly expressed in the liver, which it was suspected to be related to the metabolism. Finally, *mapk1* gene for bioinformatics analysis were selected, and the result showed that the sequence length was 2,424 bp, and there was one coding region of the predicted gene structure. Besides, divergent amino acid changes (Val-Ile at 321, 325) by comparing amino acid sequences with seven other fish were found (Figure 4A).

**TABLE 1:** Differential expression in tissues of six genes involved with salinity-alkalinity adaptation.

Gene name	Gene id	Differential expression	
		Up	Down
*cat.*	c67479.graph_c0	TYbrain_vs_TYliver, TYheart_vs_TYliver, TYkidney_vs_TYliver	TYliver_vs_TYspleen
*abcc1*	c64618.graph_c0	TYbrain_vs_TYliver, TYheart_vs_TYliver, TYkidney_vs_TYliver	TYliver_vs_TYspleen
*gpx4*	c37617.graph_c0	TYbrain_vs_TYliver, TYheart_vs_TYliver, TYkidney_vs_TYliver	TYliver_vs_TYspleen
*gpx1*	c69480.graph_c0	TYbrain_vs_TYliver, TYbrain_vs_TYkidney, TYbrain_vs_TYspleen	TYheart_vs_TYbrain
*mapk1*	c61646.graph_c0	TYbrain_vs_TYkidney, TYheart_vs_TYkidney, TYkidney_vs_TYliver	TYkidney_vs_TYspleen
*aqp1*	c26278.graph_c0	TYbrain_vs_TYliver, TYbrain_vs_TYkidney, TYbrain_vs_TYspleen	TYheart_vs_TYbrain

### Candidate genes involved with low temperature adaptation

**FIGURE 5:**
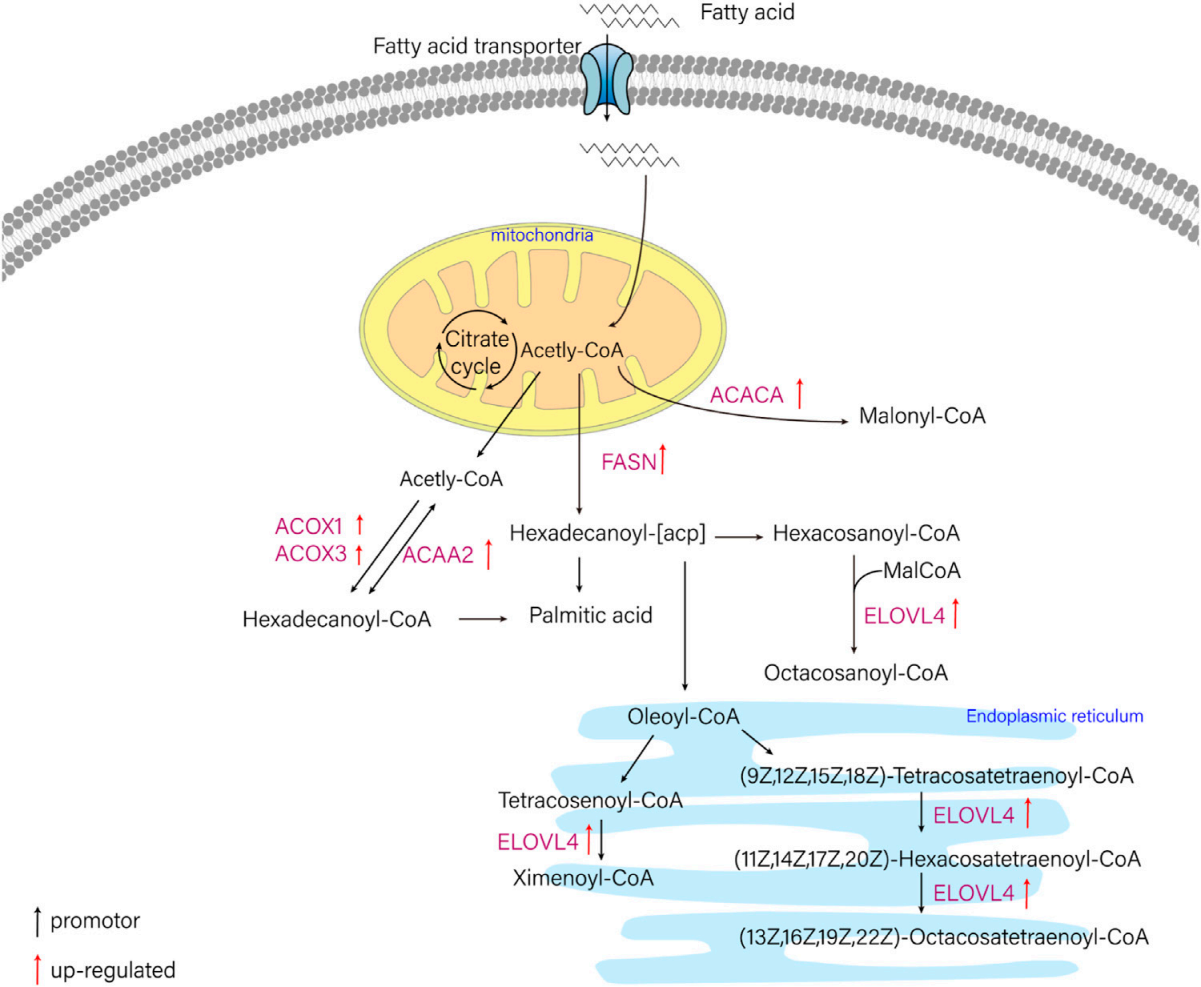
The schematic diagram of fatty acid metabolism pathway. ELOVL4 (elongation of very long chain fatty acids protein 4), ACACA (acetyl-CoA carboxylase/biotin carboxylase 1), FASN (fatty acid synthase, animal type), ACAA2 (acetyl-CoA acyltransferase 2), ACOX1 (acyl-CoA oxidase 1), ACOX3 (acyl-CoA oxidase 3).

In this study, six genes involved in fatty acid metabolism pathway, *elovl4*, *acaca*, *fasn*, *acaa2*, *acox1*, *acox3*, were selected to analyze the expression differences of six genes among liver and other tissues of *T. yarkandensis.* The results demonstrated that the expression of *elovl4*, *acaca*, *fasn*, *acox1* and *acox3* in liver were higher than that in brain, heart, kidney and spleen. The expression of *acaa2* gene in liver were higher than that in brain, heart and spleen. The expression of six genes in fatty acid metabolism pathway differed among different tissues (Table 2). These genes involved in fatty acid metabolism pathway were shown in Figure 5. The expression of six genes in liver were higher than that in other tissues, indicating that *T. yarkandensis* had relatively fast fatty acid metabolism, which might be related to its low temperature adaptation. Finally, *acox1* gene for bioinformatics analysis were selected, and the result showed that the sequence length was 5,277 bp, and there were two coding regions of the predicted gene structure. Besides, divergent amino acid changes (His-Gln at 402) by comparing amino acid sequences with seven other fish were found (Figure 4B).

**TABLE 2:** Differential expression in tissues of six genes involved in fatty acid metabolism.

Gene name	Gene id	Differential Expression			Pathway
		Up	Down	Normal	
*elovl4*	c66861.graph_c0	TYbrain_vs_TYliver, TYheart_vs_TYliver, TYkidney_vs_TYliver	TYliver_vs_TYspleen		Fatty acid metabolism
*acaca*	c67624.graph_c1	TYbrain_vs_TYliver, TYheart_vs_TYliver, TYkidney_vs_TYliver	TYliver_vs_TYspleen		
*fasn*	c63120.graph_c0	TYbrain_vs_TYliver, TYheart_vs_TYliver, TYkidney_vs_TYliver	TYliver_vs_TYspleen		
*acox1*	c66268.graph_c1	TYbrain_vs_TYliver, TYheart_vs_TYliver, TYkidney_vs_TYliver	TYliver_vs_TYspleen		
*acox3*	c67445.graph_c0	TYbrain_vs_TYliver, TYheart_vs_TYliver, TYkidney_vs_TYliver	TYliver_vs_TYspleen		
*acaa2*	c66476.graph_c2	TYbrain_vs_TYliver, TYheart_vs_TYliver	TYliver_vs_TYspleen	TYkidney_vs_TYliver	

## Discussion

Transcriptomics is a laboratory technique that explores gene transcription and its regulation in specific cells or tissues at the RNA level (Wang et al., 2009). Transcriptome analysis might be able to obtain almost all transcriptional information of a specific tissue or organ. With the development of modern molecular biology and instruments, RNA-Seq technology developed rapidly and has become an indispensable tool for analyzing differential gene expression at the transcriptome level. In recent years, it has been extensively used in fish research (Wang et al., 2009; Palstra et al., 2012; Petzold et al., 2013; Min et al., 2018). The current study attained 102.11 Gb transcriptome clean data and 100,112 unigenes by sequencing, assembling and annotating transcripts. In addition, several pathways were associated with signaling molecules and interaction, signal transduction in the top 20 signaling pathways by KEGG enrichment pathway analysis.

The expression of transcriptome usually varies in different species, tissues and organs. For instance, analyzing the gill and liver transcriptome of *Oryzias melastigma*, the sensitivity of gill and liver to salinity hypotonic was strikingly different (Pl et al., 2021). In this study, the gene expression and differentially expressed genes in tissues were analysised. The GO annotation of differentially expressed genes in five tissues had resemblance in molecular function, cell components and biological processes. In addition, the KEGG pathway enrichment analysis showed that the DEGs in five tissues is noticeably enriched including glycerolipid metabolism, MAPK signaling pathway, calcium signaling pathway, adrenergic signaling in cardiomyocytes and neuroaction ligand-receptor interaction. Ultimately, both liver and kidney were associated with metabolism as indicated by the enrichment of these pathways. Furthermore, cells in the brain were involved in regulation and signal transduction, while the heart had the most cardiomyocytes, which were bound up with cell adhesion, tight junction and cardiomyopathy. In addition, the spleen was a lymphocyte with hematopoietic and immune functions (Huth and Place, 2013; Li et al., 2015; Wang et al., 2022e; Huang et al., 2022).

In the previous researches, *mapk1* gene was an important gene in cell signal transduction process, and under the condition of high saline alkali concentration, the expression of *mapk1* gene in *T. yarkandensis* would increase remarkably (Chen et al., 2020). *Abcc1* gene was involved in the key regulatory processes of salinity and alkalinity adaptation, including ion transport and acid-base balance (Wang et al., 2022a). In the transcriptome study of *Luciobarbus capito*, *gpx1*, *gpx4* and *cat* genes participated in the regulation process of salinity-alkalinity adaptation in liver and kidney (Zhang, 2020). The role of *aqp1* gene in osmotic pressure regulation was closely related to salinity (Li et al., 2016). In the current study, *mapk1*, *abcc1*, *cat*, *gpx1*, *gpx4* and *aqp1* candidate genes involved in salinity-alkalinity adaptation were identified and in five tissues were differentially expressed in *T. yarkandensis*. Previous studies have found that the metabolism of unsaturated fatty acids in fish was sensitive in cold. Fatty acid metabolism was important for fish to survive at low temperatures, such as *Larimichthy crocea*, *Cyprinus carpio*, *Chanos*, *Ctenopharyngodon Idella* (Farkas et al., 1980; Hsieh et al., 2003; Hsieh and Kuo, 2005; Qian and Xue, 2016). Tissue distribution of transcription for 29 lipid metabolism-related genes in *Takifugu rubripes* demonstrated that in fatty acid β-oxidation, *acox1* and *acox3* gene expression levels were higher in liver than in other tissues (Xu et al., 2020). The expression levels of *acaa2* gene, related to fatty acid metabolism, would change when affected by certain environment (Yang et al., 2020). *Elovl4* gene in fish was significant in fatty acid synthesis and low-temperature stress adaptation (Liu et al., 2022), while *acaca* gene and *fasn* gene in liver were related to cold stress resistance in zebrafish (Wang et al., 2022b). In the current research, the expression levels of *elovl4*, *acaca*, *fasn*, *acaa2*, *acox1* and *acox3* genes in liver were higher. This not only showed that fatty acid metabolism of *T. yarkandensis* was relatively fast, but also indicated that the stability and fluidity were maintained by cell membranes, which might be related to its adaptation to low temperature environment. This study also provided a molecular basis for exploring salinity-alkalinity and low temperature adaptation of *T. yarkandensis*.

## Conclusion

Overall, the sequencing results of this study add new information on the gene sequence data of *T. yarkandensis*. In fact, data of the gene expression and differential expression genes analysis among five tissues were obtained. Furthermore, our findings indicated that candidate genes respectively involved in the salinity-alkalinity and low temperature adaptation were differentially expressed in diffierent tissues of *T. yarkandensis*, including *mapk1*, *abcc1*, *cat*, *gpx1*, *gpx4*, *aqp1*, *elovl4*, *acaca*, *fasn*, *acaa2*, *acox1* and *acox3*. Our study also showed that these genes were significant in salinity-alkalinity and low temperature adaptation. These transcriptome data can also be conducive to comprehensively study the population adaptive evolution and genetic diversity of *Triplophysa* family, as well as the development, protection and utilization of *T. yarkandensis.*

## Data Availability

The datasets presented in this study can be found in online repositories. The names of the repository/repositories and accession number(s) can be found below: https://www.ncbi.nlm.nih.gov/bioproject/PRJNA894312.
