# Supplementary material for: Transcriptome analysis and gene expression analysis related to salinity-alkalinity and low temperature adaptation of Triplophysa yarkandensis
Source: Front Genet. 2023 Jan 12;13:1089274. doi: 10.3389/fgene.2022.1089274 (PMC9877283; doi:10.3389/fgene.2022.1089274)
Supplement: Supplementary file 1 [file DataSheet1.docx]

**Table S1**

Statistics of sample sequencing data evaluation of *T. yarkandensis*.

| Sample | Read Number | Base Number | GC Content | %≥Q30 |
| --- | --- | --- | --- | --- |
| TYbrain1 | 20,752,474 | 6,186,590,130 | 45.19% | 93.95% |
| TYbrain2 | 21,050,671 | 6,279,527,432 | 45.01% | 93.39% |
| TYbrain3 | 22,096,627 | 6,574,463,976 | 44.90% | 93.78% |
| TYheart1 | 23,410,276 | 6,968,817,934 | 45.62% | 94.33% |
| TYheart2 | 20,538,530 | 6,116,984,120 | 45.87% | 94.34% |
| TYheart3 | 22,296,018 | 6,635,027,158 | 46.04% | 94.23% |
| TYkidney1 | 21,575,459 | 6,412,576,414 | 45.54% | 92.02% |
| TYkidney2 | 21,843,651 | 6,518,546,984 | 45.66% | 91.28% |
| TYkidney3 | 20,358,232 | 6,082,710,734 | 47.60% | 92.55% |
| TYliver1 | 25,822,968 | 7,681,805,176 | 46.04% | 94.09% |
| TYliver2 | 27,646,149 | 8,235,823,390 | 45.85% | 93.59% |
| TYliver3 | 22,029,734 | 6,588,458,184 | 45.37% | 94.21% |
| TYspleen1 | 22,148,898 | 6,603,648,552 | 46.19% | 93.90% |
| TYspleen2 | 28,770,020 | 8,592,076,262 | 45.60% | 93.87% |
| TYspleen3 | 22,231,081 | 6,632,127,118 | 46.00% | 93.74% |

**Table S2**

Statistics of *de novo* assembly of transcriptome of *T. yarkandensis*.

| Length Range | Transcript | Unigene |
| --- | --- | --- |
| 200-300 | 29,045 | 26,443 |
| 300-500 | 35,776 | 28,029 |
| 500-1000 | 40,409 | 20,766 |
| 1000-2000 | 48,675 | 10,948 |
| 2000+ | 107,663 | 13,925 |
| Total Number | 261,585 | 100,112 |
| Total Length | 587,961,701 | 108,156,316 |
| N50 Length | 3,938 | 2,579 |
| Mean Length | 2247.69 | 1080.35 |

**Table S3**

| Database categories | Number of annotated genes | Proportion of annotated genes  (%) |
| --- | --- | --- |
| COG | 5207 | 22.63 |
| GO | 19535 | 84.91 |
| KEGG | 19637 | 85.35 |
| KOG | 15404 | 66.95 |
| Pfam | 18696 | 81.26 |
| Swiss prot | 13941 | 60.59 |
| TrEMBL | 19259 | 83.71 |
| eggNOG | 18421 | 80.06 |
| NR | 19270 | 83.75 |
| Total | 23008 | 100.00 |

The statistical results of gene annotation in databases of *T. yarkandensis*.

**Table S4**

Number of differentially expressed genes between tissues of *T. yarkandensis*.

| DEG_Set | All_DEG | up-regulated | down-regulated |
| --- | --- | --- | --- |
| TYbrain_vs_TYkidney | 6473 | 2376 | 4097 |
| TYbrain_vs_TYliver | 9814 | 3818 | 5996 |
| TYbrain_vs_TYspleen | 9195 | 3855 | 5340 |
| TYheart_vs_TYbrain | 8593 | 5046 | 3547 |
| TYheart_vs_TYkidney | 2542 | 1074 | 1468 |
| TYheart_vs_TYliver | 5858 | 2615 | 3243 |
| TYheart_vs_TYspleen | 3714 | 1534 | 2180 |
| TYkidney_vs_TYliver | 2724 | 1381 | 1343 |
| TYkidney_vs_TYspleen | 1887 | 1001 | 886 |
| TYliver_vs_TYspleen | 6880 | 3610 | 3270 |

**Table S5**

Twenty KEGG pathways with most unigenes involved of *T. yarkandensis*.

| Number | Pathway | Pathway ID | Unigenes with pathway annotation |
| --- | --- | --- | --- |
| 1 | Neuroactive ligand-receptor interaction | ko04080 | 591 |
| 2 | Calcium signaling pathway | ko04020 | 497 |
| 3 | MAPK signaling pathway | ko04010 | 481 |
| 4 | Endocytosis | ko04144 | 428 |
| 5 | Focal adhesion | ko04510 | 367 |
| 6 | Regulation of actin cytoskeleton | ko04810 | 355 |
| 7 | Herpes simplex virus 1 infection | ko05168 | 355 |
| 8 | Salmonella infection | ko05132 | 342 |
| 9 | Ribosome | ko03010 | 315 |
| 10 | Cell adhesion molecules | ko04514 | 299 |
| 11 | Adrenergic signaling in cardiomyocytes | ko04261 | 297 |
| 12 | Phagosome | ko04145 | 296 |
| 13 | Apelin signaling pathway | ko04371 | 294 |
| 14 | Tight junction | ko04530 | 289 |
| 15 | Wnt signaling pathway | ko04310 | 282 |
| 16 | Carbon metabolism | ko01200 | 277 |
| 17 | Protein processing in endoplasmic reticulum | ko04141 | 277 |
| 18 | Cytokine-cytokine receptor interaction | ko04060 | 255 |
| 19 | mTOR signaling pathway | ko04150 | 238 |
| 20 | Vascular smooth muscle contraction | ko04270 | 237 |


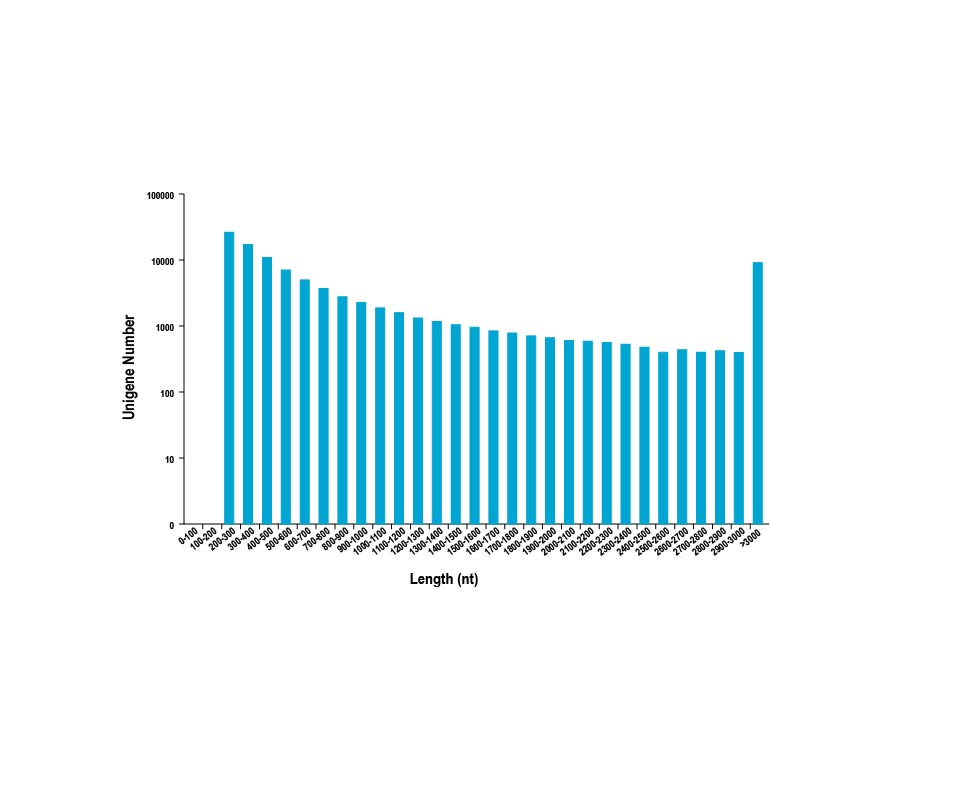


Figure S1. The statistical assembly results of unigenes of *T. yarkandensis*.
